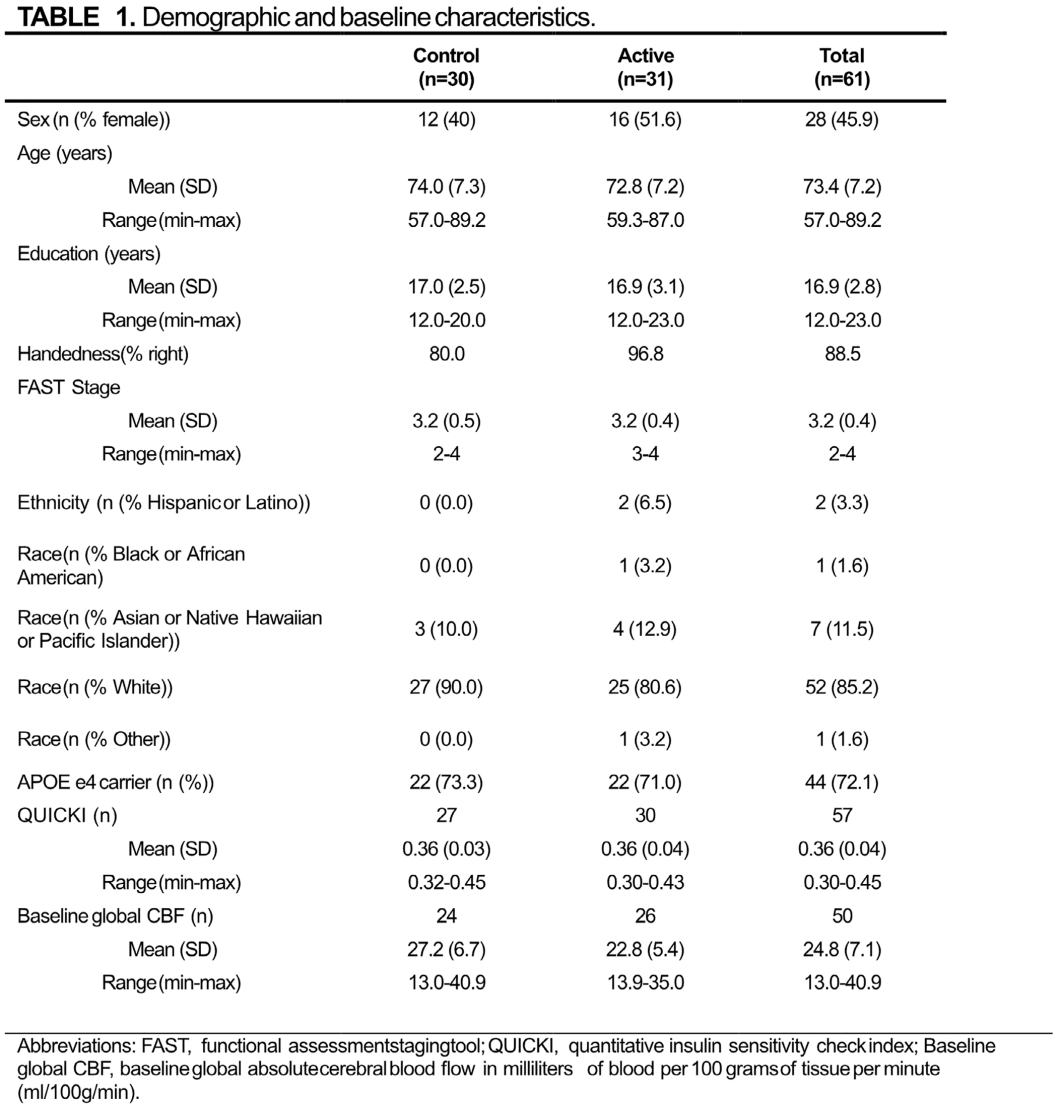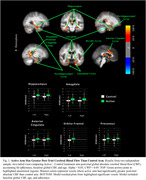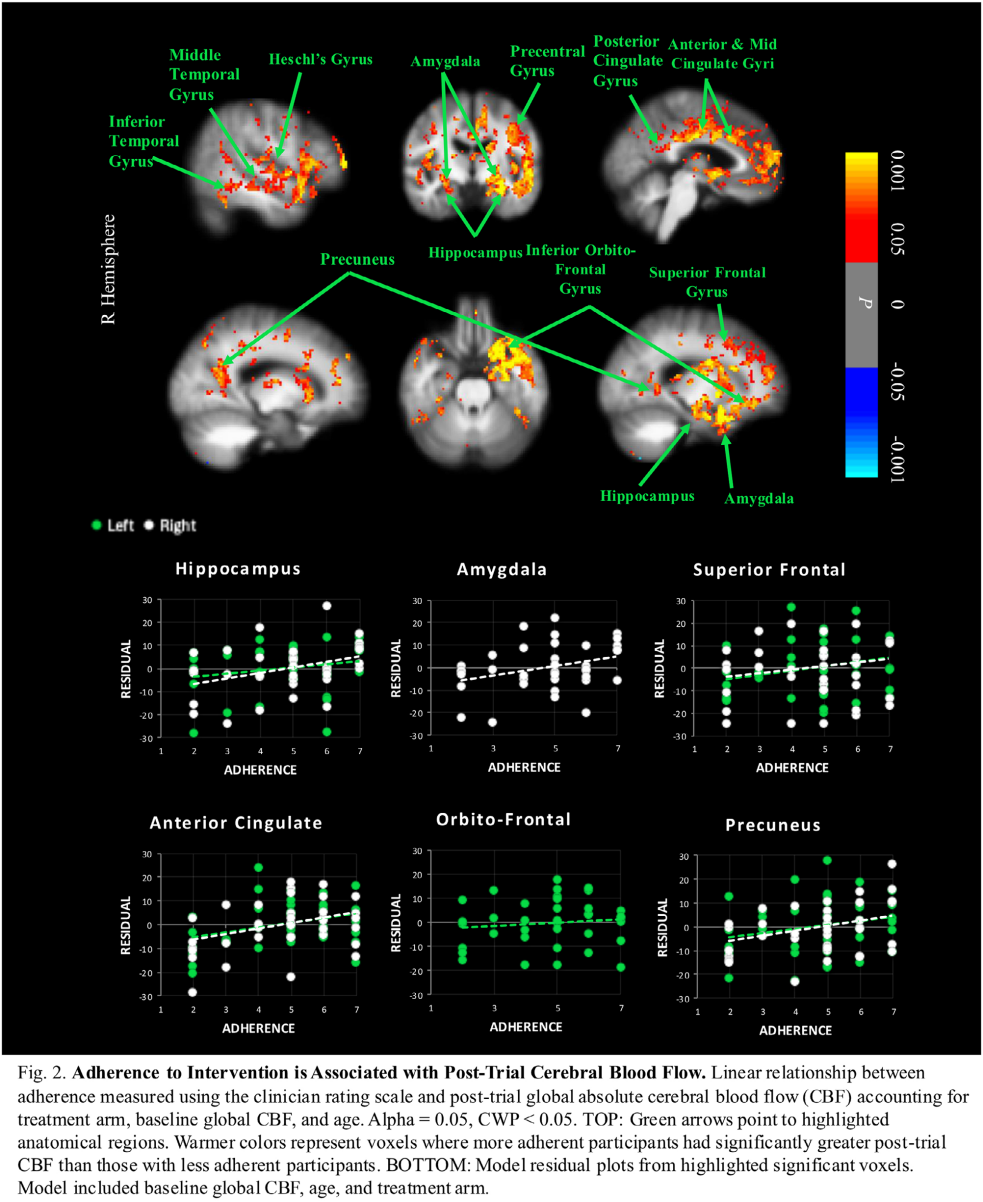# A Multi‐Modal Medical Management and Lifestyle Randomized Clinical Trial Increases Cerebral Blood Flow and Lowers Diabetic Risk in Persons with Early Alzheimer's Disease: Results from The [PREVENTION] Trial

**DOI:** 10.1002/alz70860_099523

**Published:** 2025-12-23

**Authors:** Jennifer E. Bramen, Prabha Siddarth, Emily S. Popa, Gavin T Kress, Molly K. Rapozo, Aarthi S. Ganapathi, John F. Hodes, William Sparks, Ynez M. Tongson, Andrea M Torres, Colby B. Slyapich, Ryan M. Glatt, Kyron P. Pierce, Karen J. Miller, Shannel H Elhelou, Verna R. Porter, Claudia L. Wong, Mihae Kim, Stella E. Panos, Tess Helen Bookheimer, Susan Y. Bookheimer, Cyrus A. Raji, Lee Hood, Jared C. Roach, David A. Merrill

**Affiliations:** ^1^ University of California Los Angeles, Los Angeles, CA, USA; ^2^ Saint John's Cancer Institute at Providence Saint John's Health Center, Santa Monica, CA, USA; ^3^ Pacific Brain Health Center, Pacific Neuroscience Institute and Foundation, Santa Monica, CA, USA; ^4^ David Geffen School of Medicine at University of California Los Angeles, Los Angeles, CA, USA; ^5^ The Icahn School of Medicine at Mount Sinai, New York, NY, USA; ^6^ Pacific Brain Health Center, Pacific Neuroscience Institute Foundation, Santa Monica, CA, USA; ^7^ University of Southern California Department of Psychology, Los Angeles, CA, USA; ^8^ Drexel University College of Medicine, Philadelphia, PA, USA; ^9^ Providence Saint John's Health Center, Santa Monica, CA, USA; ^10^ Mallinckrodt Institute of Radiology, Washington University in St. Louis, St. Louis, MO, USA; ^11^ Institute for Systems Biology, Seattle, WA, USA

## Abstract

**Background:**

Medical and lifestyle management are crucial for Alzheimer's disease (AD). Cerebral blood flow (CBF), vital for brain health, is reduced in AD, and influenced by modifiable risk factors.

**Method:**

The PREVENTION study is an ongoing randomized clinical trial (McEwen, 2021). Forty‐eight participants with biomarker evidence of amyloidosis had completed the study at the time of the analysis (Table 1). The control arm received recommendations and medical management for a year; the active arm additionally received coaching, exercise training, and supplementation. We examined the effects of the 1) the intervention and 2) adherence on diabetic risk (QUICKI) and post‐trial CBF measured using ASL MRI. Adherence was measured using the clinician rating scale (CRS), which uses a scale of 1‐7 (Kemp, 1998). We hypothesized that those in 1) the active arm and 2) with higher intervention adherence would have improved post‐trial QUICKI and CBF, particularly in regions previously shown to be influenced by physical activity, insulin sensitivity, and cardiovascular risk and relevant to AD (Hoscheidt, 2017; Kapasouri, 2022; Hafdi, 2022; Moore, 2015; Yang, 2023; Chen, 2011). Absolute CBF was estimated using MRI‐Cloud. Post‐trial CBF was analyzed using a linear model, accounting for baseline CBF, adherence, age, and education. Adherence was treated as a continuous measure. Change in QUICKI was analyzed using mixed effects general linear models, including treatment group, adherence, time, and the interactions between time and treatment group and time and adherence as independent variables, controlling for age. Adherence was a categorical measure, based on a cutoff score ≤ 4 (occasional reluctance) (Kemp, 1996).

**Result:**

Treatment arms did not differ in any demographic measures at baseline or CRS. The active arm (*n* = 18) showed significantly greater post‐trial CBF in regions related to cardiovascular, diabetic, and AD risk, compared to control (*n* = 20) (Figure 1; Table 2). Higher adherence scores were associated with greater improvement in regional CBF (Figure 2) and QUICKI *F*(1,55) = 6.19, *p* = 0.02).

**Conclusion:**

In this small sample, we found evidence that a multi‐domain intervention focused on medical management, exercise, and a carbohydrate‐restricted diet improved diabetic risk and CBF in patients with AD.